# Differential Capacitance Spectroscopy for Real-Time Monitoring of RNA Amplification

**DOI:** 10.1021/acs.jpcb.5c01815

**Published:** 2025-09-01

**Authors:** Steffane Q. Nascimento, Rodrigo M. Iost, Thiago C. Oliveira, Erika R. Manuli, Geovana M. Pereira, Ester C. Sabino, Frank N. Crespilho

**Affiliations:** † São Carlos Institute of Chemistry (IQSC), University of São Paulo (USP), São Carlos, SP 13560-970, Brazil; ‡ Institute of Chemistry (IQ), University of São Paulo (USP), São Paulo, SP 05508-000, Brazil; § Institute of Tropical Medicine, Faculty of Medicine, University of São Paulo (USP), São Paulo, SP 05403-000, Brazil

## Abstract

RNA amplification is central to viral diagnostics, yet current optical and fluorometric methods, such as PCR and RT-qPCR, remain costly, complex, and resource-intensive. Here, we introduce differential capacitance spectroscopy (DCS) as a real-time electrochemical method for RNA detection using loop-mediated isothermal amplification. By applying sinusoidal currents to flexible carbon fiber electrodes (0.05 cm^2^, 1 mm apart), DCS monitors changes in electrode–electrolyte interface capacitance, generating a unique arm-shoulder diagram (ASD) for RNA amplification. The ASD reveals exponential and polynomial capacitance variations, distinguishing amplified RNA under isothermal conditions. Our method eliminates the need for sophisticated instrumentation and reduces diagnostic costs while enabling rapid, high-sensitivity detection. Validated with COVID-19 patient samples, DCS provides a promising platform for affordable, scalable, and real-time RNA-based diagnostics, positioning itself as a transformative tool for disease surveillance and clinical applications.

## Graphical abstract

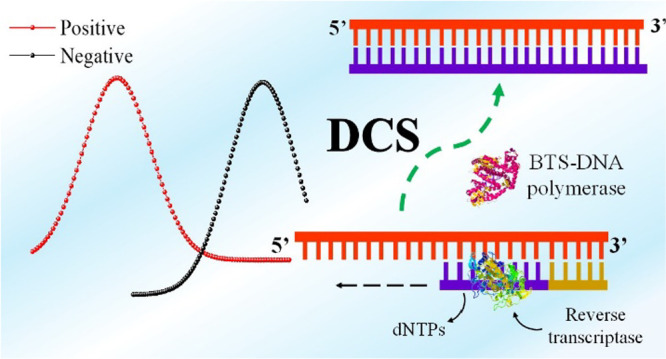

## 1 Introduction

RNA detection based on RNA amplification has emerged as an essential tool for the accurate diagnosis of viral infections, especially when rapid, inexpensive and accurate identification is required.^1–5^ Conventional methods, such as reverse transcription polymerase chain reaction (RT-qPCR), have been widely adopted due to their high sensitivity and specificity. However, such techniques rely on complex detection systems that make these methods expensive and inaccessible. Furthermore, the specificity of these techniques requires trained personnel, limiting their accessibility in technically limited settings that hinder their large-scale application, making them unfeasible at the point of care.^6^ Given these limitations, this highlights the need for alternative diagnostic approaches that are rapid, accurate, and low-cost so that they can be applied in a variety of healthcare settings.^7,8^

Loop-mediated isothermal amplification (LAMP) has proven to be a promising alternative to traditional RNA amplification methods. Unlike PCR, LAMP is applied at a constant temperature, making it a simple and more accessible technique.^6,9^ This technique uses a set of primers that target specific regions of the RNA sequence, allowing for fast, accurate and efficient amplification. The simplicity attributed to isothermal amplification makes this technique more viable for applications in environments with limited resources.^6^ Furthermore, LAMP results can be detected visually through color changes in turbidity. However, although colorimetric RT-LAMP offers a promising approach for qualitative (binary) detection, its reliance on visual readouts limits quantitative resolution and introduces subjectivity in result interpretation. These limitations can hinder high-throughput screening, particularly during large-scale outbreak testing, where automation, reproducibility, and analytical accuracy are critical.^10^

To address these challenges, we propose a novel method for monitoring LAMP amplification using differential capacitance measurements.^11^ This innovative approach leverages the changes occurring at the electrode–electrolyte interface during RNA amplification to provide real-time, label-free monitoring. By analyzing the interference in sinusoidal waves of differential capacitance, our method offers a direct and efficient way to track the amplification process.^11^ At the heart of this approach is the differential capacitance spectroscopy (DCS) technique, which is sensitive to changes in the solution interface or electrode materials. DCS provides a robust platform for detecting biochemical events associated with RNA amplification and represents a significant advancement in impedance-derived capacitance spectroscopy.^12–14^ This new technique offers several advantages over existing methods. It is low-cost, fast, and has a simpler setup based on an interfacial measurement, facilitating large-scale implementation of the method. In addition, the real-time monitoring capability of DCS increases the sensitivity and specificity of RNA detection, making it suitable for several diagnostic applications. Although the adoption of electrochemical detection systems to detect RNA amplification is a new approach, this method shows promise, and its demand has been growing increasingly in literature, mainly because it offers the development of accessible and low-cost tools for disease diagnosis, especially in low-resource settings, where traditional methods face significant barriers.^15^

Using clinical samples from COVID-19 patients, we verified our methodology and demonstrated its efficacy and utility for the diagnosis of SARS-CoV-2.^10,12,15,16^ The results presented demonstrate how DCS can be used as a general amplification technique for several viruses. This strategy can be crucial in managing public health problems that require access to rapid, accurate and affordable tests. This methodology marks a significant advance in the creation of diagnostic technologies that combine high performance, accessibility and simplicity.

## 2 Method

### 2.1 Preparation of FCF-Based Devices

Initially, the FCFs were cleaned in an ultrasound bath for 5 min with a mixture of isopropyl alcohol and ultrapure water. After the cleaning process, the FCF electrodes with an area equal to 0.0511 cm^2^ are mechanically miniaturized and placed in the 3D printed electrochemical device and are then electrochemically activated in basic aqueous solution using chronoamperometry (1.5 V (150 s) and −1.0 V (150 s), with subsequent washing in ultrapure water and dried under vacuum.

### 2.2 Preparation of Real Samples

The collection and use of patients’ saliva were approved by the Ethics Committee (CONEP-B-16) for diagnostic analysis under the Brazilian platform CONEP (Comissão Nacional de Ética em Pesquisa). Saliva samples (1 mL) were collected in sterile tubes, with care taken to avoid contamination from food or liquids. The sample volume was selected to ensure adequate nucleic acid concentration and to maintain consistent electrochemical signal quality during assay development and clinical validation. To reduce viscosity and facilitate handling, saliva was diluted 1:1 in saline solution. Cell lysis and RNA release were achieved either by heating at 95 °C for 5 min or by using a lysis buffer containing detergents. Subsequently, RNA was extracted using the phenol–chloroform method and resuspended in 50 μL of RNase-free water after purification. Although LAMP can be performed directly from crude lysates, the use of RNA purification at this stage aimed to reduce variability and potential interference in the differential capacitance signal, ensuring reproducibility during method validation.

The RT-LAMP mixture included specific primers, dNTPs, MgCl_2_, betaine, and the required enzymes (reverse transcriptase and DNA polymerase). Amplification was initiated by adding 2 μL of extracted RNA and incubating the mixture at 65 °C for 30–60 min. Results were assessed by both visual inspection (color change) and the electrochemical method proposed in this work. All procedures were conducted using sterile materials to prevent contamination, and experimental conditions were optimized according to sample quality.

### 2.3 CDS Analyses

CDS analyzes consist of three steps:

1. Preparation of the assays: Preparation of the reaction (Solution containing reagents for amplification: Master Mix (100 μL), Prime Mix (90 μL), RNA (10 μL). Then the device is submerged in this solution and is maintained at 65 °C isothermally.
2. Electrochemical Experiments: With the device stabilized at 65 °C, a series of sinusoidal current frequencies (range 10^5^–5 × 10^–2^ s-1) are applied using the EIS (Electrochemical Impedance Spectroscopy) technique. an open circuit potential (OCP) with an amplitude of 10 mV generating an imaginary impedance graph (*Z*′ vs *Z*″) which is transformed into an imaginary capacitance graph (*C*′ vs *C*″) by the following equation:

$$C=C^{\prime }-jC^{\prime \prime }$$

where “*j*” = imaginary unit, the square root of −1; Zc = −*j*/(*C*) = capacitive impedance, which can be expressed in terms of capacitance *C* and angular frequency.

3. Data processing: Application of the Second derivative in the *C*′ vs *C*″ graph, the d*C*′ vs *C*′ graph, reveals the variation of the imaginary capacitance with the angular frequency, this variation indicates how the amplification reaction occurs with the time, with this variation a positive or negative result for RNA amplification is reached.

### 2.4 Interpretation of the Results

The electrochemical data obtained via DCS are interpreted through analysis of the shape, position, and evolution of the Arm–Shoulder Diagram (ASD) and its corresponding second-derivative spectra. These graphical signatures reflect dynamic interfacial changes at the electrode/electrolyte boundary over time and across frequencies, offering mechanistic insights into the kinetics of the RNA amplification process. In positive samples undergoing successful amplification, a time-dependent decrease in real capacitance (*C*′) is observed, accompanied by concurrent alterations in imaginary capacitance (*C*″), particularly in the low-frequency region. These changes are attributed to the accumulation of negatively charged nucleic acid products, modulation of the local dielectric environment, and acidification of the medium due to proton generation. Collectively, these factors restructure the electrical double layer, whose properties are highly sensitive to ionic strength and molecular crowding. Conversely, negative samples lacking amplification show minimal or linear variations in capacitance over time and do not display the inflection points that are characteristic of active amplification. This contrast enables both visual and computational discrimination between positive and negative samples.

To enhance detection sensitivity and temporal resolution, the second derivative of the *C*′ vs *C*″ plot is computed. Peaks or inflection points in this derivative spectrum correspond to specific frequency-dependent electrochemical responses, enabling real-time identification of amplification events. These features serve as electrochemical biomarkers of reaction progression, offering both qualitative (profile morphology) and quantitative (magnitude of capacitance shift, rate of change, and onset time) insights. The analytical framework combines visual inspection of ASD signatures with quantitative modeling of capacitance dynamics, providing a robust, label-free platform for real-time RNA detection. Detailed experimental procedures are provided in the Supporting Information.

## 3 Results and Discussion

We present a three-step process for utilizing DCS. In step 1, we designed a device with two conductive flexible carbon fiber (FCF) electrodes, each with an area of 0.05 cm^2^ and spaced 1 mm apart (Figures S1 and S2), to perform sequential RNA amplification detection using DCS (Figure[Fig fig1]A–D). The technique involved applying a series of sinusoidal current frequencies to the electrodes (Figure[Fig fig1]E), which generated a set of differential capacitances on the imaginary axis of a *C*′ versus d*C*″ spectra. By utilizing the first derivative at each selected frequency, a differential d*C*″ diagram was generated, and the d*C*′ versus *C*″ diagram was obtained after amplification reaction. In the conventional capacitance diagram, the horizontal axis represents capacitance (*C*′), and the vertical axis represents imaginary capacitance (*C*″). The relationship between capacitance (*C*), imaginary capacitance (*C*″), and frequency (*f*) can be described by the equation *C* = *C*′ – *jC*″, where “*j*” represents the imaginary unit, the square root of −1. The capacitive impedance, represented by Zc = −*j*/(ω*C*), can be expressed in terms of capacitance *C* and angular frequency ω. However, in DCS, we observe that the derivative results in an arm-like and shoulder-like diagram (Figure 1F), which will be discussed in detail later. The second derivative of the *C*′ versus *C*″ curve (Figure 1E) provide additional information about the electrochemical system. For example, a positive second derivative indicates that the system has a peak in its capacitance at a certain frequency. The frequency at which the peak or dip occurs is known as the characteristic frequency of the system and can provide information about the size and structure of the system. In practical applications, analyzing the second derivative of the *C*′ versus *C*″ curve can help identify the characteristic frequency of the electrochemical system, and optimize the performance and stability of the system.^12^ For example, it can be used to choose the appropriate frequency range for measurements,^13^ or to identify potential issues such as surface adsorption or double layer effects.^14^ Overall, the second derivative of the *C*′ versus *C*″ curve obtained from EIS data is an important parameter for understanding the electrochemical behavior of a system and optimizing its performance.

**Figure 1:**
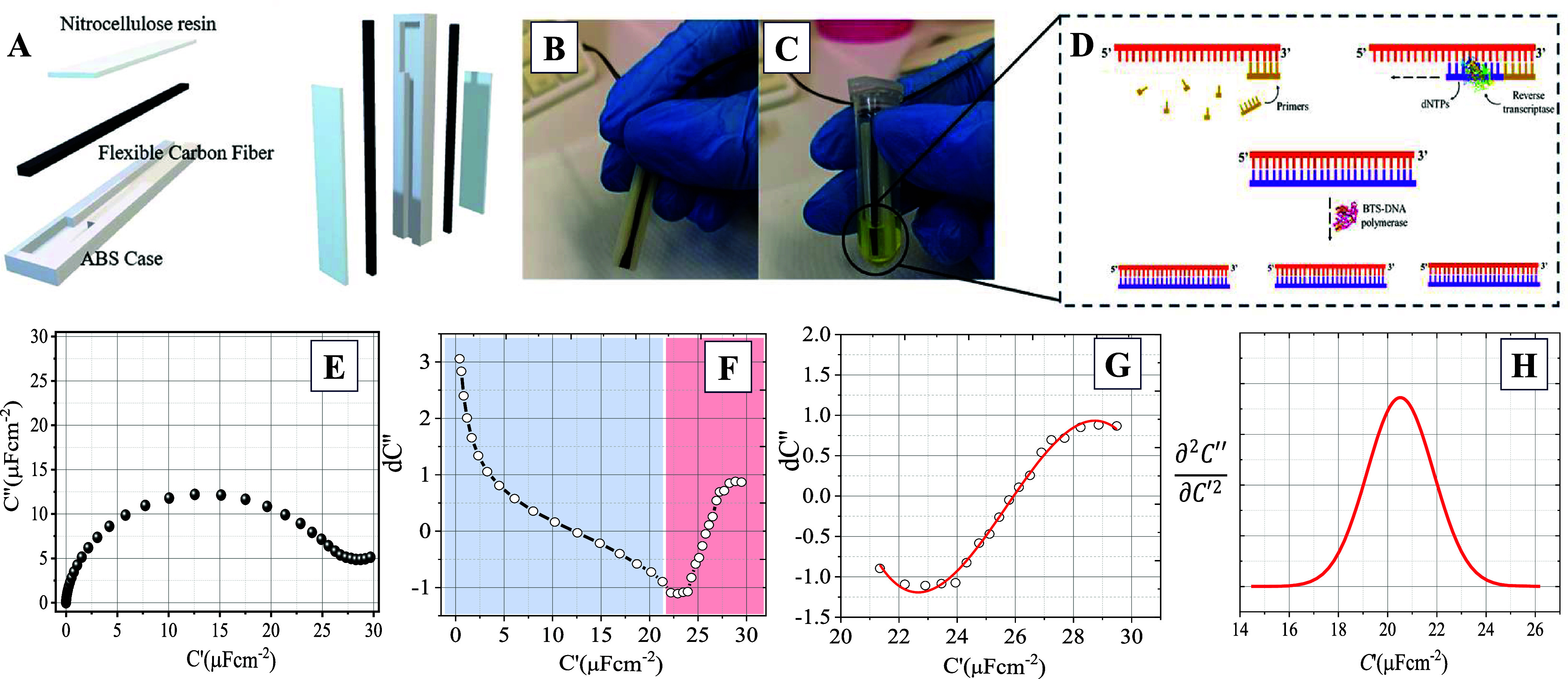
Differential capacitance spectroscopy (DCS) technique. (a) Constituents of the device and 3D image of the device vertically. (b) Photo of the real device. (c) The device after the RNA amplification reaction. (d) Illustrative scheme of RNA amplification loop reaction. (e) Spectroscopy of capacitance (*C*′ versus *C*″ curve). (f) Differential d*C*″ diagram. (g) Shoulder-like diagram. (h) DSC spectrum.

In step 2, we used the LAMP for RNA amplification. This stage comprises the reverse transcriptase enzyme to synthesize a complementary DNA strand from RNA, followed by the addition of four specific primers that recognize six different regions of the target DNA (see details in S.I.). During the LAMP process, the DNA polymerase enzyme was used at a constant temperature of 65 °C, allowing the reaction to occur isothermally.^6^ For this purpose, we developed our own dry bath system for RT-LAMP reaction (Figure S3). Finally, in step 3, we monitored RNA amplification in real-time through the differential dC” diagram (Figure[Fig fig1]F), referred to as the Arm-Shoulder Diagram (ASD). This diagram allowed for differentiation of RNA amplification by using shoulder region (Figure[Fig fig1]G), where multiple copies of a specific RNA segment were detected under isothermal conditions.

The selected frequencies induce capacitance changes in the imaginary axis, resulting in a set of capacitance values, which exhibits distinct regions of high frequency and exponential capacitance variation in the arm (eq 1), and low frequency and sigmoidal capacitance variation in the shoulder (eq 2).

$$dC^{\prime \prime }=dC_{0}^{\prime \prime }+A\cdot e^{RC^{\prime \prime }}$$ 1

$$dC^{\prime \prime }=A+B\cdot C^{\prime }+C\cdot {\left(C^{\prime }\right)}^{2}+D\cdot {\left(C^{\prime }\right)}^{3}$$ 2

At each frequency in the arm region, the imaginary capacitance, *C*″, is given by the sum of a constant offset, d*C*″_0_, and an exponential function of the real capacitance, *C*′, which is modulated by a scale factor, *A*, and a decay factor, *R*. This equation shows that the imaginary capacitance at a particular frequency is determined by both the intrinsic properties of the system (represented by d*C*″_0_) and the response to a perturbation (represented by the exponential term).

The scale and decay factors determine the shape of the exponential curve (Figure[Fig fig1]H) and provide information about the dynamics of the system. The variation in imaginary capacitance in the shoulder region, d*C*″, is expressed as a polynomial function of the real capacitance, *C*′. The polynomial function includes a constant term, *A*, a linear term, *BC*′, a quadratic term, *C*(*C*′)^2^, and a cubic term, *D*(*C*′)^3^. This equation provides a mathematical description of how imaginary capacitance changes with changes in real capacitance. Coefficient A represents the baseline change in the imaginary capacitance, while the coefficient *B* describes the slope of the relationship between real and imaginary capacitances. The coefficients *C* and *D* represent the curvature of the relationship between the two capacitances, with *C* being related to the concavity of the curve and *D* related to its skewness. Overall, this equation represents a model to understand the relationship between the real and imaginary components of the impedance response of an electrochemical system. Also, the present model can be used to extract information about the underlying physical and chemical processes that contribute to the overall capacitance response. Interestingly, the d*C*″ changes over time, allowing the ASD to differentiate different stages of RNA amplification when the device is immersed in a commercial LAMP solution. This analysis enables the detection of generation of multiple copies of a specific RNA fragment under isothermal conditions. In the shoulder region of the ASD, there is a cubic relationship between d*C*″ and d*C*′, resulting in a unique profile that distinguishes it from the arm region.

For application of ADS in human saliva samples for RNA-SARS-COV-2 amplification, we developed a versatile LAMP bioreactor equipped with precise temperature control. The system combines a customized dry bath heating device and a DCS platform, offering a robust and scalable solution for point-of-care applications. The bioreactor consists of a compact dry bath system designed for laboratory adaptability. A mechanically machined aluminum heating plate (80 mm × 20 mm) with 24 perforations for 500 μL Eppendorf tubes ensures uniform heating across samples. The heating element (LJXH PTC, preset to 65 °C) is thermally insulated and controlled via a W3001 digital temperature controller (precision: ±0.1 °C), allowing real-time monitoring and accurate temperature stability. The device is housed in a portable plastic box (156 × 114 × 79 mm), providing a simple, low-cost alternative for isothermal amplification set-ups. We validated the system using COVID-19 patient samples (RT-PCR confirmed), where the combination of LAMP amplification and DCS analysis enabled rapid and sensitive detection. The analysis was performed on saliva samples collected from patients with varying symptom durations (2–7 days) and ages (16–72 years), with results validated by RT-PCR as a reference standard (Table S1). The modular design allows compatibility with various platforms, including Eppendorf microcubes, making the LAMP bioreactor adaptable to different laboratory environments. This approach represents an efficient, cost-effective tool for RNA amplification, suitable for diagnostic applications and scalable to high throughput testing scenarios

Figure[Fig fig2], shows the results of samples obtained from patients with COVID-19, confirmed by RT-PCR (Table S1). A versatile system consisting of a LAMP solution and a device immersed in it was used. This system can be easily modified and applied to different platforms, including an Eppendorf microcube, allowing its use in different laboratory environments. Figure[Fig fig2]A presents the shoulder-like diagram for a positive and negative sample for COVID-19, which shows a significant separation of capacitance values. Figure[Fig fig2]B also presents the representative ADS in red for COVID-19 patients and in blue for non-COVID-19 patients. Figure[Fig fig2]C shows the ADS for a positive sample, where it is observed that as the reaction progresses, it causes a sharp reduction in capacitance, indicating that the amplification reaction is occurring. Figure[Fig fig2]D shows the ADS for a negative sample, which shows in amplification reaction of a negative sample there is a decrease in capacitance values, however after 60 min of reaction the capacitance remains constant, unlike what happens with a positive sample where the capacitance keeps decreasing over time. Figure[Fig fig2]E displays the correlation graph between *ΔC*' and time. This graph shows more clearly that, in a reaction with a positive sample, the reaction capacitance difference increases, while in a negative sample these values increase up to a plateau where the capacitance remains constant. The physical and chemical changes that occur during the LAMP reaction, such as changes in pH and conductivity, were also evaluated. Polymerase enzyme activity can generate protons as a byproduct, which can lead to a gradual decrease in the pH of the solution over time. These changes can also impact on the electrochemical properties of the solution, including its capacitance. The capacitance of the solution also changes with changing pH. This is because the capacitance of a solution depends on the dielectric constant, which is related to the concentration of ions in the solution. As the ion concentration changes due to the LAMP reaction, the dielectric constant also changes, leading to a change in capacitance. Additionally, the samples analyzed by DCS were also subjected to comparative colorimetric analysis (Figure 2F). The progression of RNA amplification through LAMP was validated by monitoring the pH changes during the reaction using phenol red as a pH indicator. At the initiation of the reaction, the solution maintained a neutral pH of 7.4, indicated by an orange hue. As the LAMP reaction progressed, proton generation likely due to enzymatic activity during nucleotide incorporation and pyrophosphate release lowered the pH to 5.2, causing the solution to shift to a yellow color. These pH drops, observed after 60 min, reflect the biochemical amplification process as acidic byproducts accumulate, confirming successful LAMP-driven amplification. To complement the visual pH observations, UV–vis spectroscopic analysis was performed to monitor molecular changes in the LAMP solution (Figure[Fig fig2]G). The spectra revealed characteristic bands corresponding to RNA/DNA, proteins, and the phenol red indicator. The initial absorption band of phenol red under neutral conditions diminished as the reaction progressed, giving rise to a new absorption band indicative of its acidic form. This spectral shift is directly correlated with the enzymatic amplification of the target sequence, further corroborating the pH-driven confirmation of the LAMP reaction. The analysis of the UV–vis spectra, particularly the increase in absorbance at 260 nm, confirms the successful amplification of DNA, with an estimated production of 2.3 × 10^12^ double-stranded DNA molecules in 500 μL of solution. Additionally, FTIR spectroscopy (Figure[Fig fig2]H) provided molecular insights into the reaction components. Characteristic phosphate stretching bands (1250–950 cm^–1^) confirmed the presence of primers and nucleotide incorporation, a hallmark of active DNA synthesis. Broad N–H and O–H vibrational bands (3200–3500 cm^–1^) corresponding to dNTPs and betaine were observed, while the enzymatic components displayed typical Amide I (1650 cm^–1^) and Amide II (1550 cm^–1^) bands, signifying protein activity. The combined pH, UV–vis, and FTIR analyses provide molecular evidence of the LAMP reaction progression, validating successful loop-mediated amplification through both biochemical and spectroscopic signatures.

**Figure 2:**
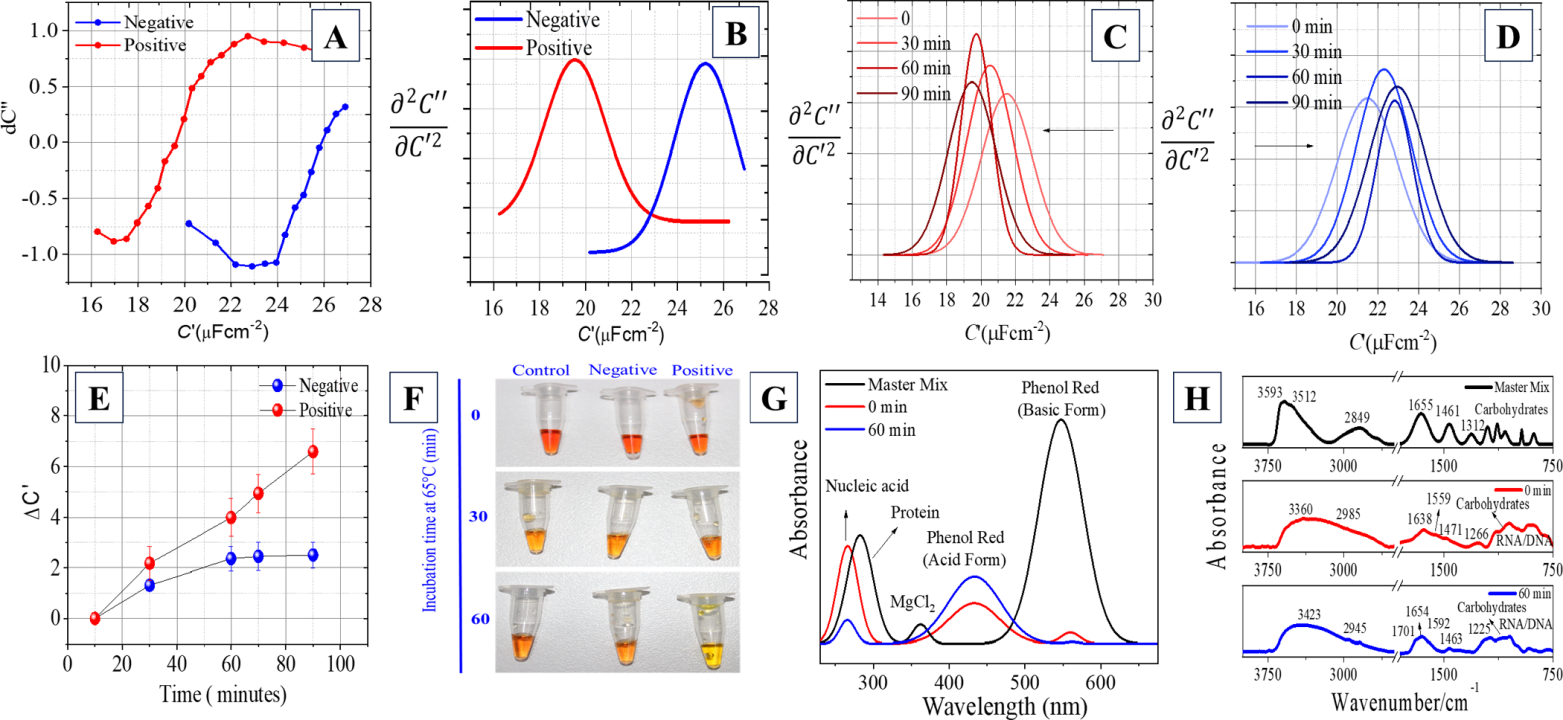
Application of ADS in human saliva samples for RNA-SARS-COV-2 amplification. (a) The shoulder plot, displayed in red for COVID-19 patients (positive) and blue for non-COVID-19 patients, illustrates a significant reduction in real capacitance among COVID-19 patients (negative). Additionally, an interference is observed, causing a lower capacitive effect across all frequency ranges. (b) The DSC spectrum, obtained through second derivative analysis of the ASD, reveals the inflection point corresponding to changes in *C*′ in red for COVID-19 patients (positive) and blue for non-COVID-19 patients (negative). (c) ADS spectrum for an amplification reaction in a positive sample. (d) ADS spectrum for an amplification reaction in a negative sample. (e) Graph of the *DC*′ × Time relationship. (f) Variation in the color of the LAMP solution as the amplification reaction progresses. (g) UV–vis spectra for the amplification reaction. (h) FTIR spectra for the amplification reaction.

The amplification kinetics of the DCS technique with LAMP reaction were analyzed and demonstrated characteristic sigmoidal behavior consistent with the expected progression of enzymatic amplification processes. Initially, a lag phase was observed, representing the preparatory stage where template RNA and primers are engaged, and enzymatic activity begins. This phase was followed by a logarithmic growth phase, where the amplification rate increased rapidly, reaching its maximum near the inflection point at approximately 21.5 min (Figure[Fig fig2]E). During this phase, exponential replication of DNA occurred, driven by the high efficiency of the LAMP primers and DNA polymerase under isothermal conditions. The amplification curve entered a plateau phase, where the reaction stabilized due to the depletion of reagents or inhibition of enzymatic activity. The rate of amplification at the inflection point was estimated to be 0.173 units/min, providing a quantitative measure of the reaction’s progression. This sigmoidal profile aligns with the expected behavior of nucleic acid amplification reactions and highlights the efficacy and reproducibility of the LAMP system under controlled conditions.

The sensitivity of the DCS system was determined to be 0.9161 units/min, calculated from the linear region of the amplification curve. The detection limit (LOD), defined as the smallest measurable capacitance change, was estimated at 0.60 units. Statistical analysis provided a 95% confidence interval for sensitivity ranging from 1.475 to 0.227 units/min, with a relative error of 12.93%. These results demonstrate the system’s ability to reliably detect small variations in capacitance during the initial phase of RNA amplification. The low LOD and high sensitivity confirm the accuracy and robustness of the proposed detection method, enabling precise real-time monitoring of nucleic acid amplification events. Since DCS system demonstrated a sensitivity of 0.9161 units/min and a limit of LOD of 0.60 units, it correlates to approximately 10–50 copies of target RNA in the reaction. This aligns well with conventional PCR, which typically detects 25–50 copies of DNA, and qPCR, which achieves performance with a detection limit as low as 2–10 copies or 2.5–10 pg of DNA/RNA per reaction. A comparative table with other LAMP-based biosensors is presented in the Supporting Information (Table S1). The analysis of saliva samples from patients aged 16 to 72 years demonstrated 100% accuracy in detecting positive and negative cases within the first 2–7 days of symptoms, as confirmed by RT-PCR, highlighting the reliability and robustness of the DCS system for early diagnosis. The cost analysis (Table S2) benchmarks our device at a unitary production cost of $2.05 USD, based on a total material expenditure of $1025.71 USD for 500 units. This positions the DCS system as a cost-competitive alternative to conventional diagnostic platforms, offering a scalable and affordable solution for large-scale deployment, particularly in resource-limited settings.

## 4 Conclusions

In summary, our results demonstrate the efficacy of the proposed DCS method as a robust alternative for RNA amplification monitoring and virus detection. By integrating DCS with LAMP, we provide a simplified, cost-effective, and reliable approach for real-time detection of viral RNA, validated here using clinical COVID-19 samples. The methodology retains the sensitivity and specificity of traditional PCR-based techniques while addressing key limitations, such as the need for thermal cycling and sophisticated instrumentation. The electrochemical detection enabled by DCS can be performed using basic equipment, such as dry bath heating systems, and generates data in a visually interpretable ASD. This eliminates the dependency on fluorescence-based readouts, expanding its applicability in resource-limited settings. The demonstrated advantages of this approach—real-time monitoring, label-free detection, and operational simplicity make it suitable for diverse applications in molecular diagnostics, including clinical disease surveillance, pathogen detection, and environmental monitoring. This work establishes a foundation for further refinement of DCS as a practical and accessible tool for RNA-based diagnostics in both research and clinical environments.

## Supplementary Material



## Notes

Complete protocols, additional figures (PDF)
