# Supplementary material for: Differential Capacitance Spectroscopy for Real-Time Monitoring of RNA Amplification
Source: J Phys Chem B. 2025 Sep 1;129(36):9051–7. doi: 10.1021/acs.jpcb.5c01815 (PMC12434665; doi:10.1021/acs.jpcb.5c01815)
Supplement: Supplementary file 1 [file jp5c01815_si_001.pdf]

Differential Capacitance Spectroscopy for Real-Time Monitoring of RNA Amplification

Steffane Q. Nascimento[1], Rodrigo M. Iost[2], Thiago C. Oliveira[1], Erika R. Manuli[3], Geovana M. Pereira[3], Ester C. Sabino[3] and Frank N. Crespilho[1]*

[1]University of São Paulo (USP), São Carlos Institute of Chemistry (IQSC), São Carlos, SP, 13560-970, Brazil.

[2]University of São Paulo (USP), Institute of Chemistry, University of São Paulo (IQ), São Paulo, SP, 05508-000, Brazil.

[3]University of São Paulo (USP), Institute of Tropical Medicine, Faculty of Medicine, São Paulo, SP, 05403-000, Brazil.

Summary

S1 Experimental Section 3

S1.1 Reagents 3

S1.2 Apparatus 3

S1.3UV-Vis analyses 3

S1.4 FTIR analyses 3

S2 Additional Results 5

S2.1Flexible carbon fiber device 5

S2.2 Dry bath heating device 7

S2.3Human Saliva Samples. 8

S.2.4 Comparative study for LAMP-based assays for SARS-CoV-2 detection 9

S2.5 Estimative Cost of FCF Devices 10

S1 Experimental Section

S1.1 Reagents

Potassium dihydrogen phosphate (KH_2PO_4), sodium chloride (NaCl), potassium chloride (KCl), sodium monohydrogen phosphate (Na_2HPO_4) were purchased from Sigma-Aldrich. Sodium hydroxide (NaOH) was obtained from Synth. The flexible carbon fibers (FCFs) were extracted from HPCCE and purchased from Delpho (Brazil). The LAMP RT - MasterMix (containing: Bioscript Pro III, Taq DNA Polymerase, RNAse inhibitor, dNTPs and Buffer and Stabilizers), Primers LAMP mix (containing: FIP/BIP 1.6μM- LF/LB 0.4 μM-F3/B3 0.2 μM) and Positive RNA control were provided by Biolinker. The water used in all experiments was obtained by a MiliQ purification system.

S1.2 Apparatus

All electrochemical measurements were performed using a μ-Autolab Type III (Metrohm Autolab, Utrecht, The Netherlands) with Nova 2.1.5. in a three-electrode and two-electrode system. The device was patterned using a Core A1 Dual 3D printer (GTMax® 3D, Brazil). Vibrational spectroscopy (micro-FTIR) was performed using a Bruker Vertex 70V spectrometer with a Hyperion 3000 microscope attached (Bruker Gmbh, Ettlingen, Germany). Micro-FTIR spectra were obtained in the transmittance mode. The FB was placed on the CaF2 window with a cooled mercury telluride and cadmium detector (MCT). Spectroscopy measurements in ultraviolet/visible region (UV-vis) were performed using a spectrophotometer Jasco V-760 between 190 and 900 nm with a quartz microcuvette (1.0 cm optical path length).

S1.3UV-Vis analyses

UV-Vis analyzes were carried out at three times, 0 min, 30 min and 1 hour. All spectra were acquired from 900 to 190 nm, 0.1 nm interval, and 100 $nm \cdot s^{-1}$ rate, single accumulation, with automatic switching of the light sources.

S1.4 FTIR analyses

FTIR analyzes were carried out at three times, 0 min, 30 min and 1 hour of reaction. For this purpose, the FCFS was placed on the CaF_2 window with a cooled mercury telluride and cadmium detector (MCT). The spectra were recorded from an average

of 128 accumulations with a spectral resolution of 4 cm^{-1} and a spectral window of 4000 – 600 cm^{-1} using an objective lens with 36x magnification

S2 Additional Results

S2.1Flexible carbon fiber device

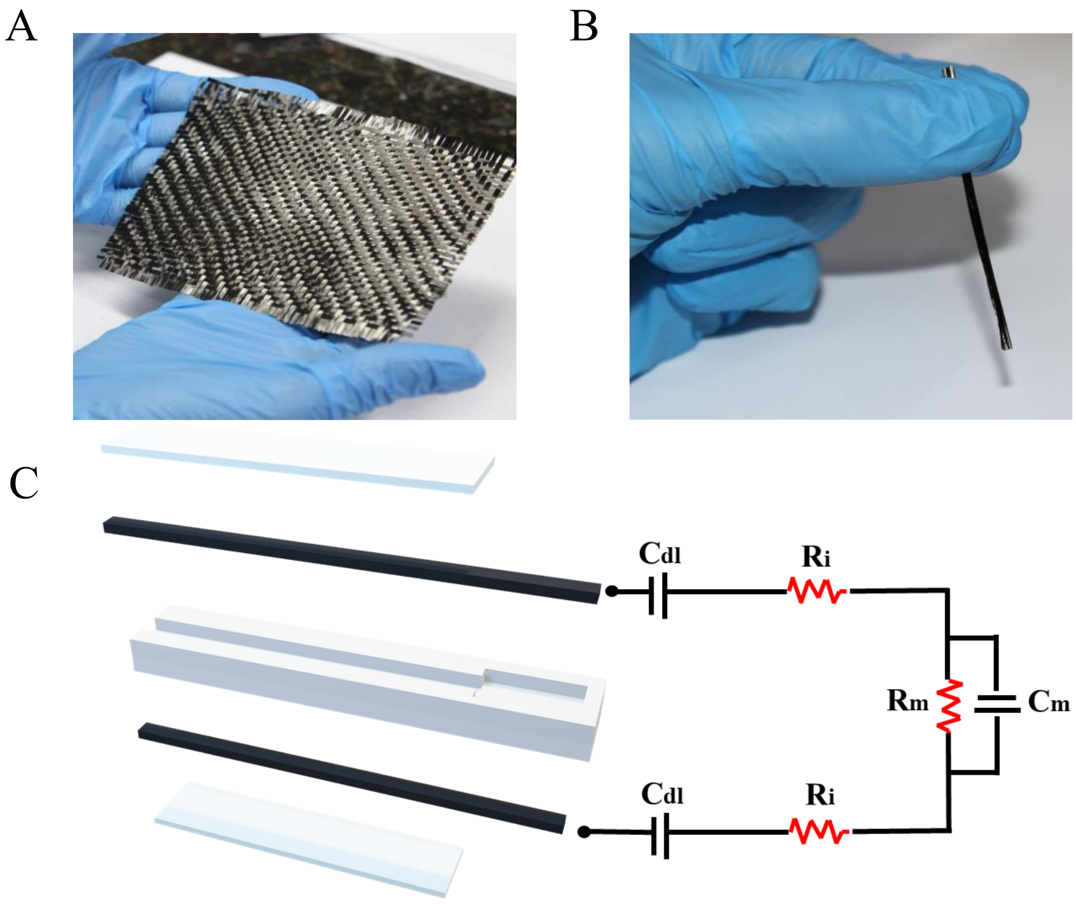

Figure S1. Flexible carbon fiber device components. A) Carbon Cloth. B) Carbon Fiber. C) Equivalent electrical circuit of the device.

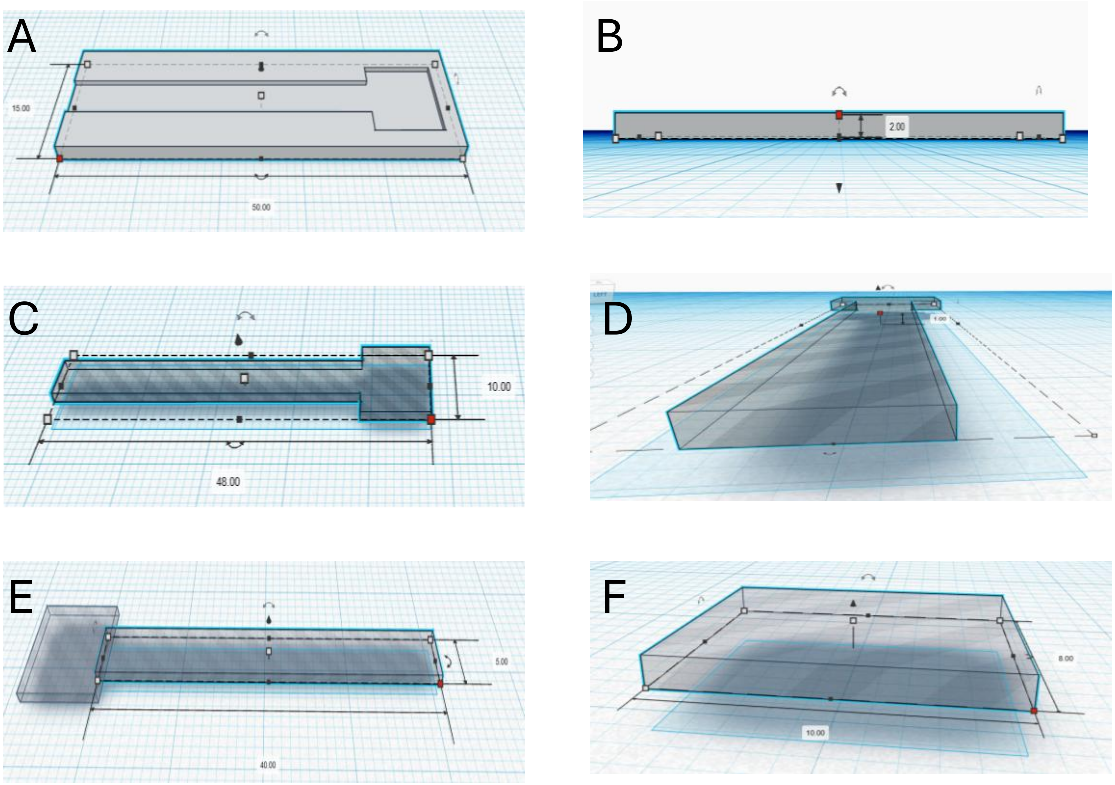

Figure S2. Device dimensions. A) Width. B) Height. C) area 1. D) area 2. E) area 3 and F) area 4.

S2.2 Dry bath heating device

The temperature control of the RT-LAMP chemical reaction was carried out with a homemade device, consisting of a plastic box with dimensions of 156 x 114 x 79 mm. The heating plate, measuring 80 mm x 20 mm and featuring a total of 24 perforations for 500 µL eppendorf tubes, was mechanically machined from aluminum to fit the dimensions of the heating box (Figure S1). The heating element, an LJXH 2PCS PTC (65 °C), supported by a thermal insulation mat, was coupled with a W3001 digital temperature controller (220 V, maximum temperature measurement range of 100 °C to 119 °C) for the construction of the device. The temperature controller used in this setup has a temperature probe attached to the heating element, displaying the measured temperature in real-time with a precision of 0.1 °C. The dry bath and heating blocks were pre-set to 65 °C prior to each RT-LAMP reaction.

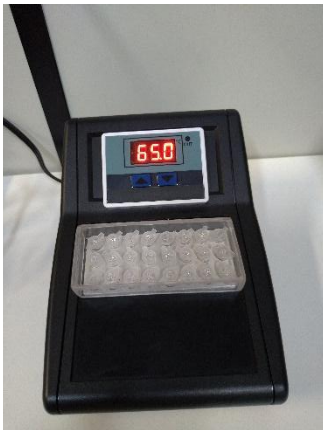

Figure S3. Homemade dry bath device for Real-time Monitoring of RNA Amplification in Patients Infected with SarsCov-2.

S2.3Human Saliva Samples.

Table S1. Human Saliva Samples.

ID SALIVA SAMPLE	SYMPTOM DAYS	AGE	PCR
240000017811	3	23	-
240000017814	2	21	+
240000017826	4	22	-
240000017834	3	28	+
240000017836	2	19	+
240000017837	4	22	+
240000017939	6	40	+
240000017935	5	24	-
240000017943	7	16	-
240000017944	2	72	-

S.2.4 Comparative study for LAMP-based assays for SARS-CoV-2 detection

Table S9 shows the comparative study between this work and other research based on isothermal amplification.

Table S2. Comparative study for LAMP-based assays for SARS-CoV-2 detection

Method	Method	Target gene	Analysis time	LOD (Copies /µL)	Reference
**RT-LAMP**	Colorimetric	ORF1ab (RdRp)	60	25	1
**RT-LAMP**	Colorimetric	ORF1a	30	50	2
**RT-LAMP**	Colorimetric	ORF1a<br>S	30	20<br>200	3
**RT-LAMP**	Colorimetric	ORF1ab	40	30	4
**LAMP**	Colorimetric	N	30	30	5
**LAMP**	Turbidity	N	35	10	6
**LAMP-DCS**	**DCS**	**S**	**30**	**10**	**This work**

S2.5 Estimative Cost of FCF Devices

The table S3 shows the materials used for the fabrication of FCF device. The cost for each material is also shown and the cost of 500 devices and their unit value were estimated.

Table S3. Estimative of cost for 500 FCF biosensor devices.

MATERIAL	QUANTITY USED (for 500 devices)	PRICE (USD)
ABS	750g	15.67
NaOH	0.4 g	0.005
RT-Lamp Kit	1u	1000
FCF	0.04088 m^2	9.81
Silicone	7 g	0.27
	**Total**	**1025.705**
	**Device unitary cost**	**2.05**

References

[1] Nawattanapaiboon, Kawin, et al. "Colorimetric reverse transcription loop-mediated isothermal amplification (RT-LAMP) as a visual diagnostic platform for the detection of the emerging coronavirus SARS-CoV-2." *Analyst* 146.2 (2021): 471-477.

[2] Rabe, Brian A., and Constance Cepko. "SARS-CoV-2 detection using isothermal amplification and a rapid, inexpensive protocol for sample inactivation and purification." *Proceedings of the National Academy of Sciences* 117.39 (2020): 24450-24458.

[3] Yan, Chao, et al. "Rapid and visual detection of 2019 novel coronavirus (SARS-CoV-2) by a reverse transcription loop-mediated isothermal amplification assay." *Clinical Microbiology and Infection* 26.6 (2020): 773-779.

[4] Lu, Renfei, et al. "Development of a novel reverse transcription loop-mediated isothermal amplification method for rapid detection of SARS-CoV-2." *Virologica Sinica* 35 (2020): 344-347.

[5] Dao Thi, Viet Loan, et al. "A colorimetric RT-LAMP assay and LAMP-sequencing for detecting SARS-CoV-2 RNA in clinical samples." Science translational medicine 12.556 (2020): eabc7075.

[6] Kitagawa, Yutaro, et al. "Evaluation of rapid diagnosis of novel coronavirus disease (COVID-19) using loop-mediated isothermal amplification." Journal of Clinical Virology 129 (2020): 104446.
